# Androgen receptor variants: another twist in the plot

**DOI:** 10.18632/oncotarget.15130

**Published:** 2017-02-06

**Authors:** Jill A. Macoska

**Affiliations:** Departments of Biological Sciences and Center for Personalized Cancer Therapy, The University of Massachusetts, Boston, MA, USA

**Keywords:** prostate, cancer, androgen receptor, mutation, metastasis

Treatment modalities targeting prostate tumors have evolved significantly since the 1970’s when diethylstilbestrol (DES) was the first line treatment for metastatic disease [1]. Contemporaneously, anti-androgen therapy in the form of cyproterone acetate demonstrated success treating advanced prostate tumors [2], and initiated a succession of hormone ablation therapies used in the 1980’s and 1990’s to combate metastatic prostate cancer. It soon became apparent, however, that prostate cancer metastases eventually failed hormone ablation therapy and exhibited a hormone refractory phenotype. To address this, therapeutic approaches aimed against non-steroidally modulated mechanisms, including the use of taxane-based drugs that targeted microtubules, DNA alkylating agents, and others, were applied [3].

Other avenues of investigation, however, continued to focus on the androgen/AR axis as the major driver of the development of castration resistant prostate cancer (CRPC). Since first identified in LNCaP immortalized prostate cancer cells in 1990 [4], mutations of the androgen receptor (AR) have been the object of thousands of studies focused on determining whether specific mutations promote prostate cancer initiation, growth, and/or progression. In addition to elucidating the biological consequences of AR mutations, several studies have identified ‘druggable’ mutations that may modulate therapeutic responses during CRPC treatment [5].

In addition to targeting the AR, some studies have focused on mechanisms governing steroid hormone synthesis. Of these, the activity of CYP17A1, which promotes intratumoral steroid synthesis, seemed a likely contender as a mechanism for persistent prostate tumor growth even under continued hormone ablation therapy. However, even the use of CYP17A1 inhibitors, such as abiraterone acetate, were not fool-proof, as some CRPC failed this line of treatment and continued to progress [6].

The elegant study by Han et al. shows that CYP17A1 treatment may select for the survival of cells harboring a ligand-binding domain mutation of the AR, Q784*, which encodes a C-terminal truncated form of AR protein. Moreover, AR Q784* mutants may function to enhance the activity of non-mutant AR under very low androgen conditions such as those encountered under hormone ablation therapy for CRPC, thus accounting for the continued survival of prostate tumor cells even under combined hormone ablation and CYP17A1 inhibitor treatment. Clearly, studies like this are crucial for understanding why some patients fail therapy - and how to negotiate the many ways that aggressive prostate cancer cells respond to, and sometimes prevail over, our best attempts to derail them.
